# Region-specific *Foxp2* deletions in cortex, striatum or cerebellum cannot explain vocalization deficits observed in spontaneous global knockouts

**DOI:** 10.1038/s41598-020-78531-8

**Published:** 2020-12-10

**Authors:** Bastiaan H. A. Urbanus, Saša Peter, Simon E. Fisher, Chris I. De Zeeuw

**Affiliations:** 1 Department of Neuroscience, Erasmus MC, 3000 DR Rotterdam, The Netherlands; 2 Language and Genetics Department, Max Planck Institute for Psycholinguistics, Nijmegen, The Netherlands; 3 Donders Institute for Brain, Cognition and Behaviour, Radboud University, Nijmegen, The Netherlands; 4 Netherlands Institute for Neuroscience, KNAW, 1105 CA Amsterdam, The Netherlands

**Keywords:** Social behaviour, Behavioural genetics

## Abstract

*FOXP2* has been identified as a gene related to speech in humans, based on rare mutations that yield significant impairments in speech at the level of both motor performance and language comprehension. Disruptions of the murine orthologue *Foxp2* in mouse pups have been shown to interfere with production of ultrasonic vocalizations (USVs). However, it remains unclear which structures are responsible for these deficits. Here, we show that conditional knockout mice with selective *Foxp2* deletions targeting the cerebral cortex, striatum or cerebellum, three key sites of motor control with robust neural gene expression, do not recapture the profile of pup USV deficits observed in mice with global disruptions of this gene. Moreover, we observed that global *Foxp2* knockout pups show substantive reductions in USV production as well as an overproduction of short broadband noise “clicks”, which was not present in the brain region-specific knockouts. These data indicate that deficits of *Foxp2* expression in the cortex, striatum or cerebellum cannot solely explain the disrupted vocalization behaviours in global *Foxp2* knockouts. Our findings raise the possibility that the impact of *Foxp2* disruption on USV is mediated at least in part by effects of this gene on the anatomical prerequisites for vocalizing.

## Introduction

Studies of a large three-generation British family (the KE family) identified a missense mutation of the human *FOXP2* gene causing a severe speech and language disorder^1^. Affected individuals in this family present with deficits in sequencing of oral movements, as well as impaired performance in expressive and receptive language tests^2^. Although rare^3^, multiple independent point mutations disrupting *FOXP2* have since been reported in additional cases of speech/language disorder^4–6^. In line with these mutations, several different types of mouse models of *Foxp2* dysfunction have since been generated and used to investigate impacts on vocal and motor behaviours (reviewed by French and Fisher^7^). Such mouse models include *Foxp2* knockouts^8,9^, as well as point mutations mirroring those found in cases of human speech disorder, most notably the *Foxp2-R552H* mutation which matches the etiological *FOXP2 R553H* mutation of the KE family^10,11^. Despite the variability in genetic approach, these mice display a number of consistent features. Homozygous mice that completely lack functional *Foxp2* typically have lifespans of approximately 3 weeks and are smaller than wild type littermates, with global developmental problems that include delays in neural development and neurite growth^8,10–13^. Conversely, mice with heterozygous *Foxp2* mutations survive to adulthood, but often show abnormalities at both the cellular and systems physiological level; including altered induction and/or expression of neuronal plasticity in several brain areas^11,14^, as well as reduced performance in sensorimotor association and motor-skill learning tasks^14,15^.

A common area of focus for studies of these *Foxp2 *mutants/knockout mice has been vocal behaviours, in particular the generation of ultrasonic vocalizations (USVs)^9–11,16–20^. USVs are short (~ 20 ms), high-pitched calls (between 60 and 120 kHz), which are produced by mice at different stages of development, both as pups and in adulthood^21^. Pup USVs, which are typically clustered in short sequences, have been shown to induce retrieval behaviours by the mouse dam, highlighting an important functional role^22^. Indeed, as young mice are blind, deaf, and poikilothermic, isolation from the nest can be a hazardous predicament^23^. One of the main factors driving the generation of USVs during isolation is thought to be hypothermia^21,23,24^. Hypothermia leads to an increase in vocalizing behaviours and subsequently also anxiety^23,24^, thereby raising the possibility of using USVs as a measurable read-out for emotional cognitive states. Because USVs can provide a behavioural indicator of an internal trait and also help to assess effects of gene disruptions on social communication, analysis of USVs has become a popular research tool in mouse studies.

So far, much of the research into potential effects of *Foxp2* disruption on mouse vocalization has focused on pup calls, and on lines with global mutation or knockout of the gene, i.e., where the gene is disrupted constitutively in all cells^9–11,18,20^. Across the different models, call rates were highly reduced in the homozygous mutant/knockout animals, and somewhat affected in heterozygous mice, although the latter was inconsistent across different studies (see French and Fisher^7^ for further discussion). Other spectral characteristics such as pitch, syllable use, and other variables were generally less affected, or completely intact. Interestingly, non-ultrasonic distress calls, which can be elicited with a tail-pinch, have been reported to be unaffected even in homozygous mutants^11^. One limitation common to all these studies is that they were based on global *Foxp2* disruption, that is mutation or inactivation of the gene in every cell of the animal. Thus far, few studies have looked at the impact of *Foxp2* interference within particular neural circuits on USVs using conditional knockouts; USV phenotypes have been identified in adult mice presenting with cerebral cortex-specific *Foxp2* disruption, with pups presenting with only subtle changes in vocalizing behaviours^25,26^. Moreover, pup USVs have been shown to be affected by *Foxp2* sumoylation within cerebellar Purkinje cells^27^. Thus, when substantive effects on vocalization have been observed, it has remained unclear which neural circuit(s) may be responsible, and even whether the effects are brain-related or due to peripheral factors involving Foxp2 activity in other tissues.

Here, we investigated three different types of conditional *Foxp2* knockout lines^28^ in which the gene was inactivated in distinct parts of the brain, including a cerebral cortex-specific knockout (*Emx1-Foxp2*), a striatum-specific knockout (*Rgs9-Foxp2*), and a cerebellar Purkinje cell-specific knockout (*L7-Foxp2*). These regions and neuronal subpopulations were chosen based on data on the normal neural expression patterns of the gene^29^. We compared the region-specific knockout animals to mice with a global, spontaneous deletion of *Foxp2,* as well as to wild type littermates. We hypothesized that the region-specific *Foxp2* knockouts would impact properties of pup USVs, as the relevant neural circuits have previously been shown to be involved in motor coordination, including call production. For example, the cerebellum is known to coordinate many different types of motor performance^30,31^, and a *Tsc1* mutation specific to cerebellar Purkinje cells has been shown to affect USVs^32^. Likewise, previous research has indicated that the primary and secondary motor cortices as well as the anterodorsal striatum probably also contribute to the generation of USVs in mice^33,34^. However, we found that none of the region-specific *Foxp2* knockouts led to a clear deficit in the generation of pup USVs, whereas animals with a spontaneous global deletion of *Foxp2* showed prominent differences from wild type littermates. Interestingly, pups with spontaneous *Foxp2* deletion showed an overproduction of noise clicks at moments when they were expected to generate USVs, suggesting a possible co-involvement of other body structures, such as the lungs and/or cartilage in the generation of USVs.

## Results

### Pup vocalization rates are largely unaffected in region-specific knockouts

**Figure 1:**
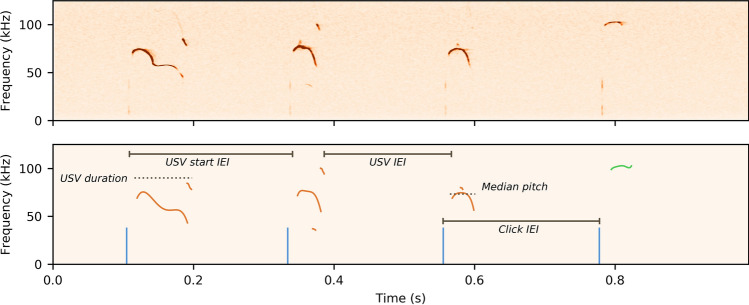
A visual representation of the analysis of the USV recordings. Top: an example trace from a healthy mouse. Bottom: the same data after extraction and processing. Multi-component vocalizations are indicated in brown, and simple vocalizations are indicated in green. Blue vertical lines are the locations of clicks. We calculate three forms of inter-event intervals (IEIs): USV end to USV start IEIs, USV start to USV start IEIs and click IEIs.

**Figure 2:**
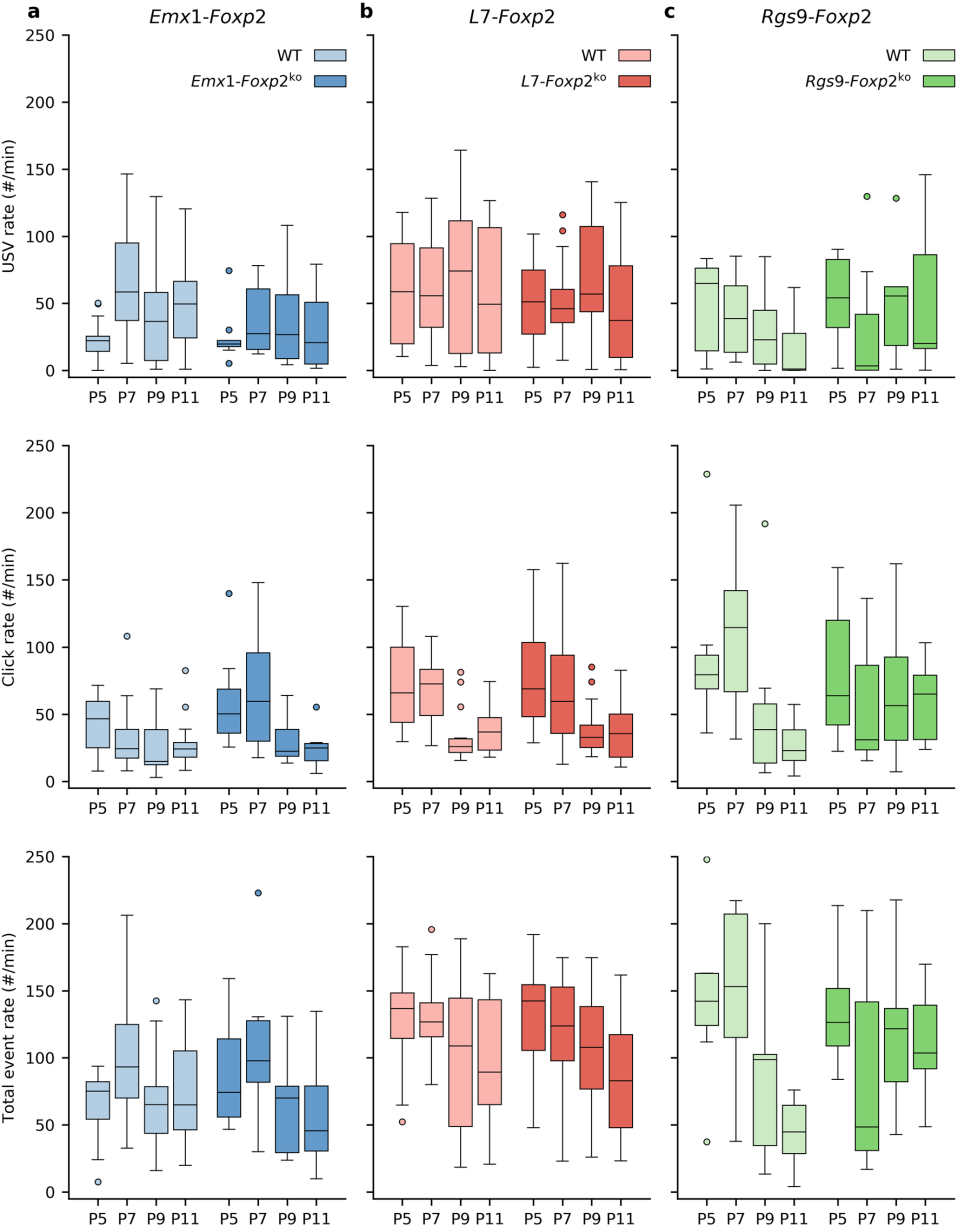
Call rates in the three conditional knockout groups are unaffected. Top row: USV rate in calls per minute for the three conditional knockout groups. WT mice and mutants are not significantly different in any of the groups. Middle row: click rate in clicks per minute. Mutant animals do not significantly differ from their WT littermates. Bottom row: event rate as the sum of USVs and clicks per minute. No effect of genotype was found on any measure for either the *Emx1-Foxp2* (**a**),* L7-Foxp2* (**b**), or *Rgs9-Foxp2* (**c**) mice. Click rates and USV rates are unaffected. In this and all following boxplots, the center line of the box indicates the median, whereas the top and bottom edges of the box indicate the 75th and 25th percentile respectively. The whiskers indicate 1.5 × IQR, and individual points are outliers. Y-axes scaling is constant for each subfigure.

To investigate whether any of the three region-specific mouse models presented with vocalization deficits we analyzed 5 min recordings from each type of conditional knockout line and their wild type littermates at four different ages (Fig. 1). In contrast to significant reductions in pup call rates observed in studies of mice with global *Foxp2* disruption^10,11,19,20^, USV rates were unaffected in all the three types of conditional knockout mice that we tested (*Emx1-Foxp2: p* = 0.48; *Rgs9-Foxp2*: *p* = 0.58; *L7-Foxp2: p* = 0.47) (Fig. 2). Likewise, neither the click rates nor the total rate of events (USVs and clicks combined) were affected (click rate: *Emx1-Foxp2: p* = 0.34; *Rgs9-Foxp2*: *p* = 0.99; *L7-Foxp2: p* = 0.99; event rate: *Emx1-Foxp2: p* = 0.97; *Rgs9-Foxp2*: *p* = 0.50; *L7-Foxp2: p* = 0.21) (Fig. 2). Subsequent analysis of other vocalization characteristics, such as pitch and duration also showed no apparent deviations from wild type littermates for either the *Emx1-Foxp2* group or the *L7-Foxp2* group (mean duration: *Emx1-Foxp2: p* = 0.48; *L7-Foxp2: p* = 0.39; median pitch: *Emx1-Foxp2: p* = 0.86; *L7-Foxp2: p* = 0.36; Supplementary Fig. 1). While the *Rgs9-Foxp2* mutant mice did not differ from wild type animals in the pitch of their vocalizations (*Rgs9-Foxp2: p* = 0.26) nor in the mean USV duration (*Rgs9-Foxp2: p* = 0.45), we did find a significant interaction between age and genotype for the USV durations (*Rgs9-Foxp2: p* = 0.04). Additional post-hoc comparisons indicated that there was only a significant difference between the *Rgs9-Foxp2* mice and their WT littermates at P11 (*p* = 0.006). As earlier research into the expression of *Rgs9* has shown that its expression is robust from at least P8^35^, we decided to perform additional analyses on a subset of the data from the *Rgs9-Foxp2* group. All previously performed analyses were repeated using only data from P9 and P11, i.e. we analysed all parameters described above using only the data from P9 and P11. We found that these analyses yielded equivalent results to the full dataset, with no significant effects found between genotype and each dependent variable. Henceforth, all data analyses for the *Rgs9-Foxp2* group are thus reported for the complete dataset, encompassing all recordings from P5 to P11. Moreover, additional attention will be given in the discussion to the temporal aspects of each promoter used here.

The pitch of USVs generally showed a bimodal distribution with one peak around 60 kHz and another peak around 90 kHz. We next investigated to what extent this distribution changed during development. USVs produced during the first week of life belonged more often to the lower peak of the distribution, and this distribution gradually shifted towards 90 kHz over time (Supplementary Fig. 2). None of the conditional knockout groups showed any obvious sign of developmental impairment in USV production capacity, as indicated by the consistent movement from lower pitched vocalizations to higher pitched vocalizations from P5 to P11 (quantified by the proportion of vocalizations over 75 kHz; *Emx1-Foxp2: p* = 0.52; *Rgs9-Foxp2: p* = 0.43; *L7-Foxp2: p* = 0.36; no significant interactions between genotype and age were found for any of the groups). Analysis of mouse weight, a parameter often affected in homozygous global *Foxp2* mouse models^8–11^, also indicated no major developmental delays for any of the mouse lines (*Emx1-Foxp2: p* = 0.63; *Rgs9-Foxp2: p* = 0.44; *L7-Foxp2: p* = 0.99). Taken together, we find that the mice in *Emx1-Foxp2* group, *Rgs9-Foxp2* group, and *L7-Foxp2* group are unaffected for all parameters tested, contrasting with phenotypes reported in global *Foxp2* knockout mouse pups^9,10,20^.

**Figure 3:**
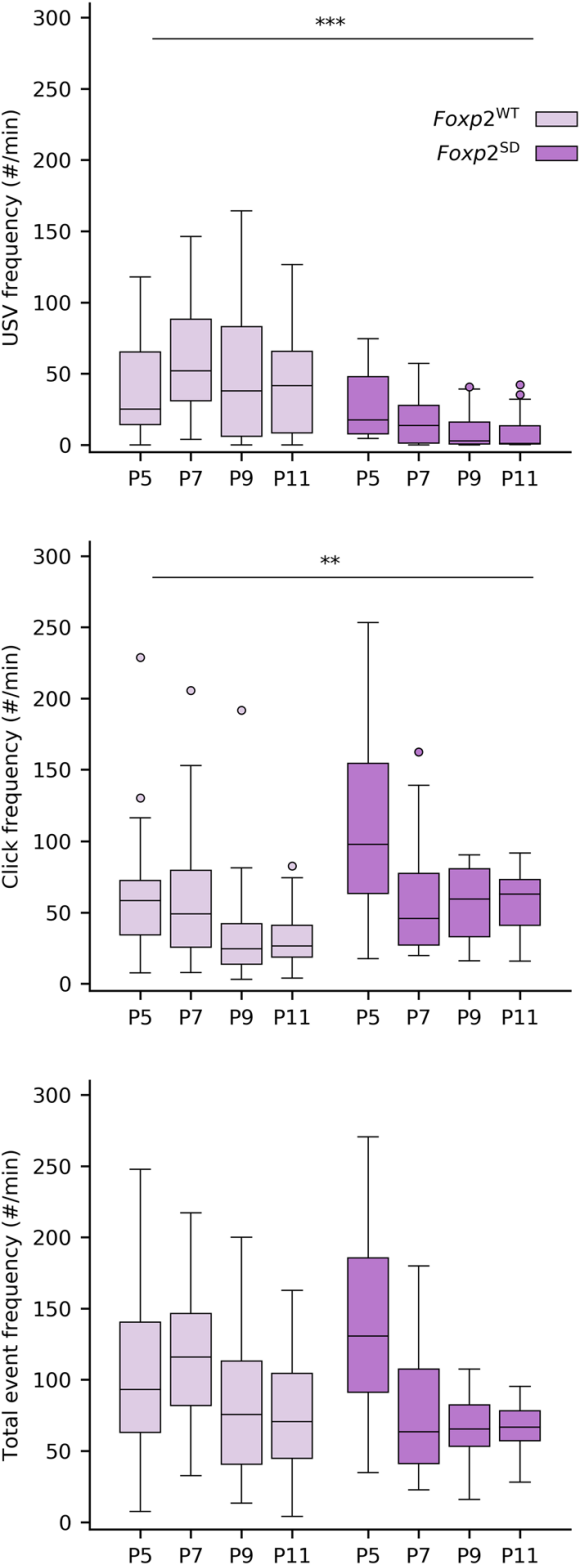
USV and click rates are affected in *Foxp2-*SD mice compared to WT mice, whereas the total event rate is mostly unaffected. Top: the USV rate is significantly reduced in the *Foxp2*-SD mice, consistent with established literature on pup USVs in mice with *Foxp2* disruption. Middle: Click rates are significantly increased in the *Foxp2-*SD mice. Bottom: comparing the sum of both USVs and clicks between the WT and *Foxp2-*SD mice only shows a difference at P7. Significance: *p < 0.05; **p < 0.01; ***p < 0.001.

Homozygous global *Foxp2* mutants show strong or complete reductions in USV call rates^10,11,19,20^, and prior studies of homozygous *Emx1-Foxp2*, *Rgs9-Foxp2,* and *L7-Foxp2* mice show differential effects on motor skills in relation to limb movements^28^. Given these earlier findings, the current lack of an obvious detrimental phenotype in the generation of pup USVs in the region-specific *Foxp2* knockout posed the question of whether our vocalization measurements were adequate. To address this point, we went on to study mice with a spontaneous *Foxp2* deletion (generated as a side-product of our breeding strategy, and designated as *Foxp2*-SD mice; see “Methods”), effectively rendering heterozygous, yet global, *Foxp2* KOs. Using the same analyses as for the other knockout groups, we found that the *Foxp2-SD* mice consistently produced fewer USVs than their wild type counterparts (*p* = 1 × 10^–5^), replicating findings found in previous analyses of mice with *Foxp2* disruptions (Fig. 3). Interestingly, the *Foxp2*-SD mice also emitted more clicks than their wild type counterparts (*p* = 0.02). Considering the event rate as the sum of the USV and click rates, we found that the overall event rate of the *Foxp2-*SD mice was not significantly different from that in the wild type mice (*p* = 0.23), raising the possibility that clicks actually represent unsuccessful efforts to produce USVs. Further post-hoc analysis of the significant interaction between age and spontaneous deletion presence (*p* = 0.01) did however indicate that there was a significant difference between wild type mice and *Foxp2-SD* mice in overall event rate at P7 only (*p* = 0.004). In addition to the differences in vocalization rates, we also found a significant interaction between age and *Foxp2-SD* for the mean vocalization duration (*p* = 0.02; significant differences found between WT and mutant mice at P7: *p* = 0.01; P9: *p* = 0.02; P11: *p* = 0.048) and median vocalization pitch (*p* = 0.003; significant difference found at P11: *p* = 1 × 10^–5^). However, the developmental trajectory of the USV pitch was not overtly affected (proportion of vocalizations above 75 kHz: *p* = 0.77; Supplementary Fig. 3). Lastly, we found that the weight of the *Foxp2-SD* mice increased less with age than their wild type counterparts (*p* = 0.025), consistent with earlier studies concerning germline *Foxp2* mutants^9^. In short, we found that none of the conditional homozygous *Foxp2* knockouts presented with a large reduction in USV production, while the spontaneous heterozygous global *Foxp2* KOs showed a phenotype in line with the altered vocalizations reported in prior studies. These findings indicate that the impact of *Foxp2* expression in the cerebral cortex, striatum as well as cerebellum is relatively limited in the context of pup USV production, and that complete inactivation of the gene in these sites is not sufficient to recapitulate phenotypic effects found in global knockouts.

### Click patterning follows USV patterning in both wild types and Foxp2-SD mouse pups

**Figure 4:**
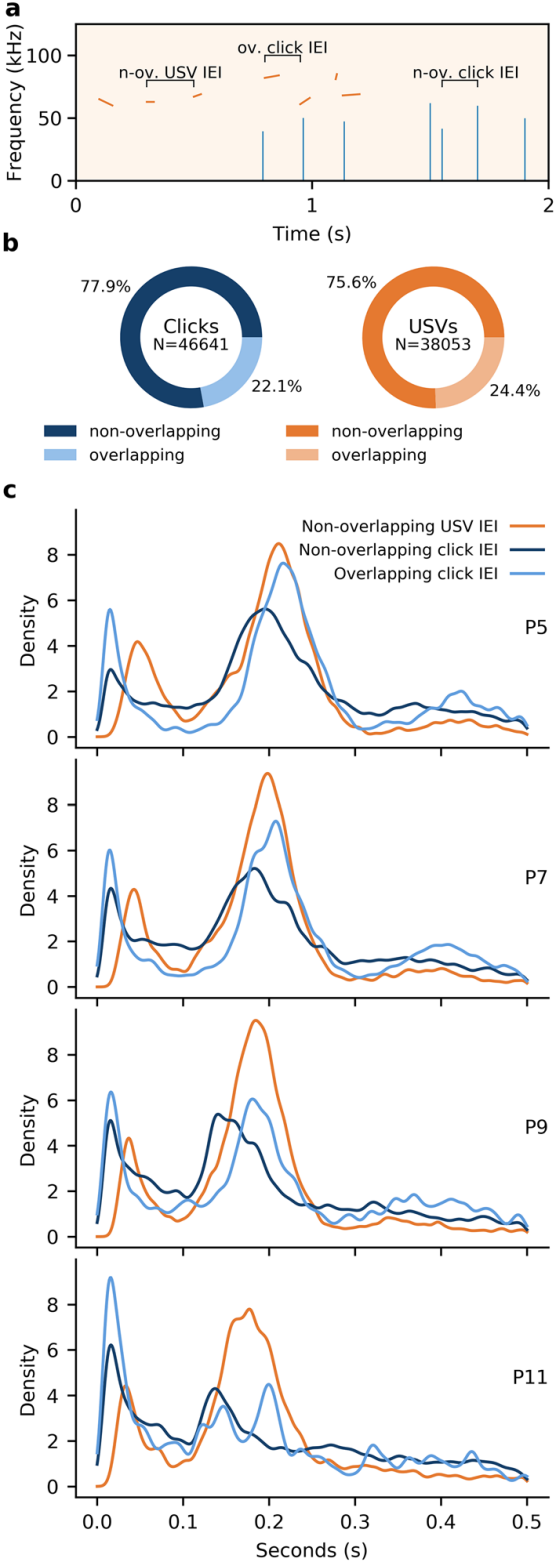
Inter-event intervals for USVs and clicks show a clear bimodal distribution. (**a**) Schematic showing the different click and USV combinations analysed here. USVs (orange) either occur alone (left grouping), or in conjunction with a click (middle grouping). Similarly, clicks either occur in conjunction with a USV (middle) or alone (right). Inter-event intervals (IEI) were calculated as shown. (**b**) Left: Of the approximately 46 thousand clicks analysed from wild type animals, 22.1% co-occurred with a USV. Right: similarly, 24.4% of the 38 thousand USVs recorded from wild type animals co-occurred with a click. (**c**) Kernel density estimation (KDE) of the non-overlapping USV IEIs show a bimodal distribution, with the first peak indicating vocalizations uttered within the same respiration, and the second peak indicating vocalizations uttered in consecutive respirations. Similarly, clicks co-occurring with USVs show a similar patterning, with a bimodal distribution indicating within-respiration and within-bout clicks.

If a click indeed represents an unsuccessful effort to produce a USV, we expect to also find a strong relation between clicks and USVs in healthy wild type animals, and to find similar patterns in the *Foxp2-*SD mice. In wild type pups, a significant portion of clicks co-occurred with USVs in that 10,324 out of the 44,641 clicks recorded in our datasets occurred in the period between 20 ms before the USV and the end of the USV (22.1%; mean overlap per recording: 21.5%). More specifically, these co-occurring clicks often coincided with the start of the vocalization; 73.4% of them occurred in the 20 ms before the USV, while 26.6% occurred during the USV itself. Likewise, 24.4% (mean overlap per recording: 25.7%) of all USVs showed at least one click co-occurring with them. Randomizing the temporal location of clicks for each recording 100 times, and calculating the average proportion of USVs with an overlapping click showed that the overlap in our data is significantly higher than that of randomized data (P5: *p* < 0.001; P7: *p* < 0.001; P9: *p* < 0.001; P11: *p* < 0.001). Along the same lines, non-overlapping clicks, i.e., clicks that do not co-occur with USVs, showed a similar distribution of their inter-event intervals (IEIs) to that of the USVs (non-overlapping click IEIs v. non-overlapping USV IEIs: P5: *p* = 0.33; P7: *p* = 0.20; P9: *p* = 0.26; P11: *p* = 0.23; non-overlapping click IEIs v. overlapping click IEIs: P5: *p* = 0.40; P7: *p* = 0.62; P9: *p* = 0.70; P11: *p* = 0.59), which remains consistent during development showing a leftward shift of the IEI over time (Fig. 4). The IEI distribution for the USVs found here is consistent with those found in previous literature^36,37^. The slight leftward shift of the second peak in the non-overlapping clicks can be explained by the fact that USVs tend to elongate respirations, thus increasing the minimum time needed between consecutive calls. In short, the patterning of clicks in wild type pups indicates a close relationship to their production of USVs, with joint developmental changes and similar sequencing. Moreover, the similarity between IEI distributions for non-overlapping clicks and USVs indicate that clicks are produced with a similar patterning as fully-formed USVs.

The *Foxp2-*SD mouse pups emitted 23,017 clicks over all recordings, 1491 (6.5%) of which co-occurred with USVs. We consider this reduction in overlap with USVs to be due to the reduction in USVs, as the percentage of USVs with overlapping clicks was equivalent to that in the wild type mice (1351/5099 USVs, 26.5%). The temporal distribution of the clicks with respect to that of the USVs in the *Foxp2-*SD mice was also equivalent to that in the wild type mice; 74.7% of the clicks occurred in the 20 ms preceding the vocalization, leaving 25.3% for the clicks occurring during the vocalization itself. Given the fact that these clicks tend to co-occur with ultrasonic vocalizations in such a stereotyped way, we consider clicks and USVs as closely related, and this relationship does not change in the *Foxp2-*SD mouse pups, despite their reduction in USVs.

### Analyses of vocalization patterns suggests alternative locus of call production deficits

The lack of a direct relation with one or more of the brain region-specific knockouts and deficits in USV production described above raises the possibility that physical abnormalities rather than neurological deficits might explain the reduced USV production of global *Foxp2* knockout mouse pups, at least with regard to the neural sites targeted in this study. If *Foxp2* expression affects the development of a physical component pertinent to USV production, such as the lungs, musculature, or cartilage^38,39^, then one might expect a disturbed relationship between the number of USVs per respiration and the number of USVs per call-sequence. To explore this possibility, we further quantified the USVs and clicks per respiration and per bout. Previous experimental findings by Sirotin et al.^37^ indicated that vocalizations separated by less than 60 ms can be considered as being produced within the same respiration, whereas vocalizations separated by more than 60 ms and less than 300 ms can be considered to be part of the same bout, defined as a sequence of vocalization-containing respirations separated from the next bout by at least 300 ms. As we hypothesized that the vocalization phenotype in the mouse pups with spontaneous germline deletions could partly be a result of non-neurological defects, we expected these *Foxp2-SD* mouse pups, but not the brain region-specific knockouts, to present with a lower percentage of respirations containing more than a single USV. Similarly, we expected to find fewer respiratory bouts consisting of more than a single USV-containing respiration. Accordingly, we also expected that these phenotypes should disappear when we consider both USVs and clicks in the respiration and bout, given the assumption that a click is an unsuccessful attempt to produce a USV.

**Figure 5:**
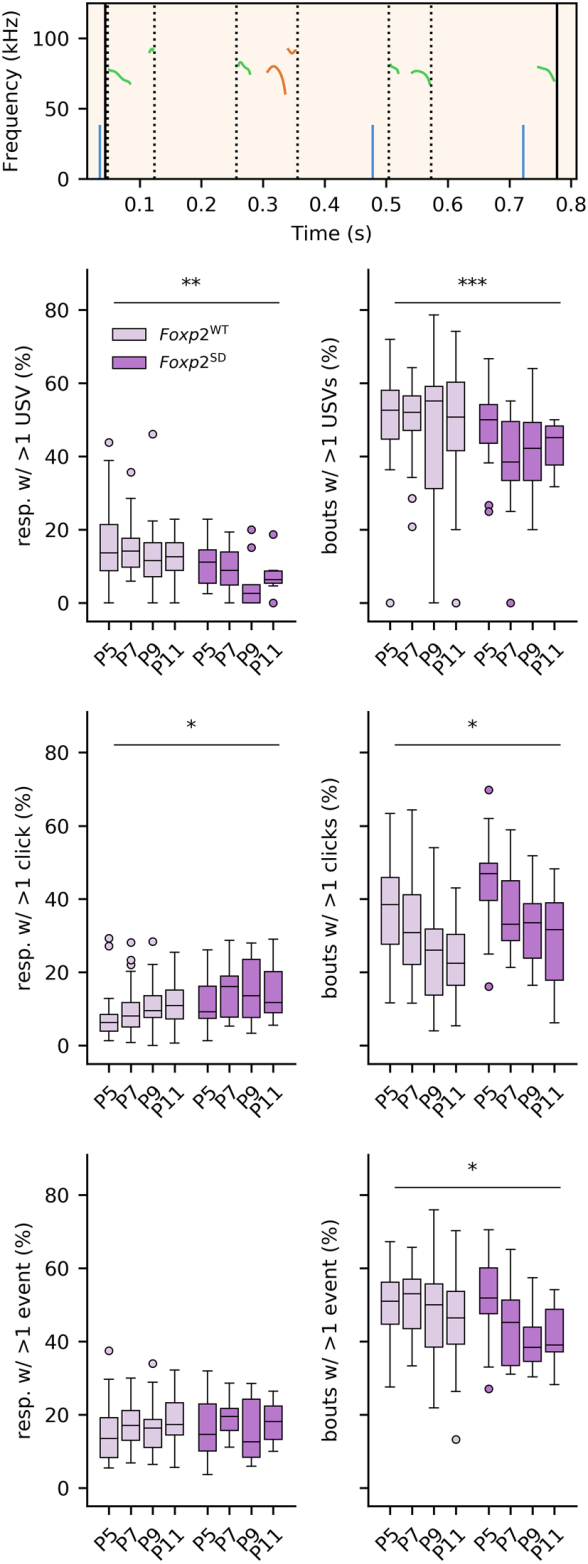
Call patterning within respirations and within bouts are affected in *Foxp2-*SD mice compared to WTs. Top: An illustration of vocalizations occurring within the same respiration (dotted vertical lines). These sets of USVs are considered to be a single unit when considering the bout sequencing of calls. The solid black lines indicate a single bout made up of three USV-containing respirations. Left column: the percentage of respirations with more than a single USV (top), the percentage of respirations with more than a single click (middle), and the percentage of respirations with more than a single event. The number of USVs within a respiration is reduced in the *Foxp2-*SD mice. The number of total events within a respiration is unaffected. Considering the sequencing of these respirations within bouts, we found that a lower percentage of bouts emitted by the *Foxp2-*SD mouse pups contain more than one USV-containing respiration, indicating an inability to produce longer sequences of USVs. In contrast to this, the percentage of bouts made up of more than one click-containing respirations is increased in the *Foxp2-*SD mutants. Lastly, we found a reduction in the overall percentage of bouts longer than one respiration in the *Foxp2-SD* mice. This reduction could be the result of USVs being more likely to be patterned into bouts, which is evident from the higher percentage of bouts with more than one USV-containing respirations. Significance: *p < 0.05; **p < 0.01; ***p < 0.001. The Y-axis scaling is the same for each subfigure.

Analysis of these parameters in the three different brain region-specific knockouts indicated no apparent deficits (percentage respirations with more than a single vocalization: *Emx1-Foxp2: p* = 0.55; *Rgs9-Foxp2*: *p* = 0.68; *L7-Foxp2: p* = 0.99; percentage bouts longer than a single respiration: *Emx1-Foxp2: p* = 0.26; *Rgs9-Foxp2*: *p* = 0.43; *L7-Foxp2: p* = 0.36). All these mice generally produced one to two vocalizations per respiration, and the bouts were mostly made up of one or two respirations, consistent with Sirotin et al.^37^. In contrast to the conditional knockout mice, the *Foxp2*-SD mice had a lower percentage of respirations containing more than one USV (*p* < 0.001) (Fig. 5). Moreover, *Foxp2-SD* mouse pups emitted fewer bouts that were longer than a single USV-containing respiration (*p* < 0.001) compared to wild type counterparts (Fig. 5). In contrast to this, we found that the sequencing of click-containing respirations into bouts was affected in the opposite direction; *Foxp2-SD* mouse pups generally emitted more bouts containing numerous respirations than wild type mouse pups (*p* = 0.038) (Fig. 5). However, the number of clicks per respiration was unaffected in these mice (*p* = 0.76) (Fig. 5). When we took both the clicks and USVs into account, we found no difference between *Foxp2-SD* mouse pups and wild type pups in the number of events per respiration (*p* = 0.44) (Fig. 5). However, the length of bouts emitted by the mutant mice did differ from wild type mice in that the *Foxp2-*SD mutants showed a lower percentage of bouts with more than one event-containing respiration (*p* = 0.02) (Fig. 5). These results indicate that *Foxp2-*SD mice are affected in their USV production. More specifically, these mice emit fewer USVs per respiration, and emit fewer bouts longer than one respiration, whilst emitting more bouts containing more than one click-containing respiration. Altogether, these findings suggest that the observed alterations in *Foxp2-SD* mouse pups may be predominantly physical in nature, as opposed to neurological. Overall, we find that the conditional *Foxp2* KO models tested here do not present with any USV phenotype, suggesting that *Foxp2*’s impact on USV production is not mediated by the cerebellum, the cerebral cortex or the striatum.

## Discussion

Here, we provide evidence that reduced USVs observed in mouse pups with global *Foxp2* disruptions cannot be explained by roles of this gene in the cortex, striatum or cerebellum, key neural sites of *Foxp2* expression as well as key sites involved in motor control in general. We show this by investigating three conditional *Foxp2* mouse knockout lines, with disruptions that individually impact each of the critical brain regions. In these conditional knockouts we only found a slight increase in the duration of USVs at one tested time point (P11) for the *Rgs9-Foxp2* (striatal knockout) mice; no other significant alterations in pup vocalizations were observed. Overall, despite homozygous loss of *Foxp2* expression from each of the targeted regions, none of the conditional lines recapitulated the significant reduction of vocalizations of homozygous (and some but not all heterozygous) *Foxp2* knockout lines reported in prior studies^9–11,20^. When we investigated mouse pups with spontaneous deletions of the *Foxp2* gene, we did observe a significant reduction in USV production, indicating that our assays are sensitive enough to detect such abnormalities. Moreover, we found that, although USV-rates were significantly reduced, click rates were increased. It has been reported in prior studies that cartilage-specific *Foxp2* knockout can induce disturbances in pup USV production^40^ and that combined loss of *Foxp2* and *Foxp1* (a close paralogue) can also disrupt lung alveolarization^12^. Thus, our findings add to the hypothesis that reduced USV production in mouse pups that lack functional *Foxp2* could partly be explained by effects on the physical machinery recruited for USV production. This possibility also aligns well with the patterning of clicks in pups with spontaneous, but not brain region-specific, deletions observed in our study. Clicks are brief broadband noises that are emitted by young mice both in concurrence with their USVs as well as in isolation. We found that a portion of clicks are patterned similarly to USVs, and could be hypothesized as an attempt to produce ultrasonic calls.

Our results are the first to show that the clicks found in vocalization recordings of mouse pups are consistently patterned like USVs. So far, only a small number of studies have described the occurrence of these clicks. Interestingly, one study of a *Foxp2-R552H* line found that homozygous mutants emitted more clicks than their heterozygous and wild type counterparts^11^. However, further interpretation of these clicks has thus far been absent. Here, we show that the clicks often co-occur with USVs, which suggests similar physiological underpinnings for these different types of vocalizations. An alternative interpretation, however, would suggest that these clicks merely reflect coincidental movements made by the pup during the USV-recordings. We consider this explanation unlikely, given our finding that isolated clicks (i.e., clicks not co-occurring with USVs) follow inter-event spacings that are similar to those of USVs. To interpret those data within the context of a coincidental movement hypothesis would suggest that these pups perform a particular non-USV-related behaviour at timing intervals similar to the emission of their calls. Additionally, mouse pups at P5 show significantly different behaviours than mice at P11, which makes it difficult to speculate on a single behaviour that is consistently performed by mouse pups of all ages. Lastly, if clicks were dependent on some interaction with the direct environment, such as the rubbing of the skin against the side of the container, then we would expect the presence and frequency of click to be dependent on the exact experimental setup in use. However, when we tested various types of containers and types of bedding for our experimental setup preceding this study, we consistently saw clicks occurring on the live spectrogram during the recordings. We thus conclude that these short, broadband clicks are closely linked to USVs. Still, additional research into the source of these clicks using techniques that allow for the investigation of the exact patterns of respiration would be prudent. One hypothesis could be that clicks are a consequence of the rapid increase in intratracheal pressure during vocalization^41^.

Two out of three conditional *Foxp2* mouse knockouts did not present with any pup vocalization deficits in the analyses we performed. The lack of a phenotype in the cerebellar PC-specific mice is particularly surprising, as it contrasts with earlier data showing that a sh-RNA-mediated knock down of *Foxp2* in Purkinje cells led to a significant reduction in the number of neonatal vocalizations^27^. A possible explanation for this, and other discrepancies, could be that this difference is due to the timing of the knockdown; the sh-RNA mediated knockdown of *Foxp2* was induced at E12.5, which thus impacts the cerebellum at an earlier timepoint than our L7 promoter. Still, this divergence in findings does hint at an issue inherent to these genetic manipulations (see later in this discussion). The *Rgs9-Foxp2* knockouts, in which the gene is disrupted in medium spiny neurons, did exhibit a relatively mild phenotype, which could potentially inform us about roles of the striatum during vocalizing behaviours. Earlier research by Arriaga et al.^33^ and Arriaga and Jarvis^34^ has implicated the anterodorsal striatum in the neural pathway supporting vocalization production. Murugan et al.^42^ showed that a shRNA knockdown of *Foxp2* in a key nucleus in the striatum of zebra finches leads to a reduction in the dopamine-related context-dependent modulation of their songs. Intriguingly, there is further evidence for the modulation of USVs in rats due to dopamine depletion^43,44^. Whether the pathways by which striatum-specific *Foxp2* loss impacts on mouse pup USVs is similar to that in zebra finches or rats is unclear, especially given the differences in the functionality of vocalizations in these animals compared to young mice. Regardless, our observations of altered USV durations in the pups with striatal *Foxp2* disruptions seem quite distinct from findings typically obtained in studies of global *Foxp2* knockouts. Possibly, the global effects of *Foxp2* loss on other tissues overpower any more specific effects of the striatum on USVs of the affected pups. Nonetheless, this result is corroborating evidence for the impact the striatum can have on vocalizing behaviours.

The lack of any major phenotypic differences in the three conditional KO lines could provide some insight into a puzzling phenomenon in *Foxp2* USV research, by way of a reinterpretation of previous data. Previous studies have shown that vocalizing behaviours in young global Foxp2 mutants are often impaired, though the exact extent of the impairments vary between studies. In particular, the rate of vocalizing is significantly reduced in most models studied^9–11,20^. Interestingly, most studies investigating adult vocalizations in heterozygous *Foxp2* knockouts report deficits that are profoundly different from those found in pups. While phenotypes in mouse pups tend to center around call rate decreases, adult vocalizations tend to predominantly be affected in their sequencing during social behaviours. Identified phenotypes in adults are a mild disturbance of the syllable usage, changes in the contextual use of vocalizations, and changes in the sequencing and rhythmicity of calls^16,17,25^. So far, this discrepancy between the phenotypes in adults and in pups has not been explained. One plausible explanation for this is that pup vocalizations are anxiety induced, whereas adult vocalizations are not. Adult mice vocalize mostly during social encounters, and in particular during mixed-sex interactions^21,45^. The differing contexts for vocalizing may thus suggest the usage of different neural circuits and this may thus modulate the impacts of a *Foxp2* mutation. However, an alternative explanation for the difference between phenotypes of young and adult mice could lie in the production of the vocalizations and the physical requirements for these calls. If the vocalization deficit is predominantly due to an inability to produce calls correctly, perhaps due to effects on both cartilage^40^, or lung tissue^12^, then these impairments are possibly more easily overcome, if not rendered irrelevant, as an adult. It may well be possible that young mice are simply more affected by relatively mild hindrances in their call production, whereas older mice may have developed past these ailments, and can thus produce calls without issue. Future experiments investigating USVs of young and old mice during prolonged periods of time, in combination with respiration assays, could help us to test this hypothesis.

Our data show that while USV rates are significantly decreased in our *Foxp2-SD* pups, click rates are increased, and could account for the “missing” USVs. This could suggest that, whilst these pups do have the intent to vocalize, physically producing USVs is difficult. Given these data, we have suggested an alternate hypothesis for the way *Foxp2* may impact pup vocalizations, namely via its impact on the anatomical prerequisites for vocalization production. However, there are additional alternative explanations for our data. Notably, there is a possibility that while the knockout of *Foxp2* in the specific brain regions tested here is not sufficient to cause the phenotypes found in global knockout mice, the combination of a number of these conditional knockouts could be capable of inducing the established phenotype. In addition, it is possible that *Foxp2* expressed in another neural circuit could underlie the phenotype found in global knockout mice, for instance the thalamus^46^, globus pallidus^47^, inferor olive^48^ or brain-stem^49^. Another factor to take into account, as with all conditional genetic interventions, is the temporal expression patterns of the various cell-specific promoters. Information concerning the timecourse of Cre-expression for each of our three promoters is limited. However, it has been established that *Emx1* expression commences around E9.5, continuing into the early postnatal days^50,51^. *L7* is thought to be ubiquitously expressed in cerebellar Purkinje cells from P9, with the caveat that *L7* expression starts around E16 in a characteristic banding pattern, suggesting that PC-specific Cre-recombinase activity may already exert its impact at a point in time preceding P9^52,53^. Lastly, information on the expression of *RGS9* is scarce. One study has established robust expression at P8 for *RGS9,* though it is important to note that expression was not checked before P8^35^. In short, these heterogeneities in cell-specific promoter-expression should be kept in mind when interpreting these data, as they could account for the discrepancy between phenotypes. The impact of *Foxp2* on pup USVs could well be predicated upon a delicate interplay between the development of particular circuits and *Foxp2*’s expression at particular points in time. Though previous data have shown the impact of the conditional *Foxp2* mutations investigated here^28^, with robust *Foxp2* impairment in the targeted regions, we cannot exclude the possibility that the USV-relevant *Foxp2* effects on development have already taken place at the time Cre-recombinase intervenes. It is important to note, however, that the lack of a significant interactions between time and genotype in our statistical analyses does suggest a limited impact of promoter expression. In short, there are limitations inherent in our genetic approach, but the current data, in combination with prior studies showing impacts of *Foxp2* on lung and cartilage development, could suggest that care should be taken with the interpretation of *Foxp2* USV studies, as it is plausible that vocalizations are impaired due to factors beyond neural impairments.

Most previous readings of *Foxp2* mouse USV data have consistently ascribed an important role for neural *Foxp2* in mediating the production of ultrasonic calls. This fits well with the ingrained representation of human *FOXP2* as a crucial gene underlying speech, following the discovery of rare mutations impairing such abilities. However, the focus on the impact of *Foxp2* on the brain and on the etiology of speech have fueled interpretations of USV data suggesting that mouse pup calls and human spoken communication are in some ways directly comparable. The lack of either a concrete neuro-anatomical model or a conceptual framework for what USVs represent has paved the road for somewhat speculative interpretations of these data. Future research should be more careful to take into account the possible effects that whole-body mutations can have on relevant non-neural regions, as they can offer alternative explanations for the assayed behaviours. Moreover, a conceptual framework concerning the function of the behaviour is necessary for proper interpretation of data. For example, previous interpretations of USV data have ascribed functional relevance to the use of different syllables in the call repertoire of mice, in that significant differences in usage are considered relevant, regardless of evidence for functional differences between syllable types. Whilst the production of different syllables presumably has a neural basis, the functional relevance of a frequency jump or a harmonic is unclear; it is important to acknowledge that the existence of these syllables could also be due to differences in lung function, or could be a side-effect of the mouse’s anatomy (consider harmonics in vocalizations). In short, we argue that more care should be taken in the interpretation of vocalization data, both at the conceptual level concerned with the meaning and functionality of USVs, as well as at the anatomical level concerned with the structures involved in the production of calls.

## Methods

### Animals

Floxed *Foxp2* mice were originally generated by French et al.^8^ All mice were bred on a C57BL/6J background. The *Foxp2* floxed mice were crossed to *Emx1-Cre, Rgs9-Cre* or *L7-Cre* mice^28,54,55^, resulting in a brain region-specific, homozygous *Foxp2* knockout for cerebral cortical pyramidal cells, striatal cells, or cerebellar Purkinje cells, respectively. Primers used for PCR characterization of the genotypes of these mice are listed in Supplemental Table 1. All experiments were conducted in line with the European guidelines for care and use of laboratory animals (Council Directive 86/6009/EEC). All experimental procedures were approved a priori by an independent animal ethical committee (DEC-Consult, Soest, The Netherlands), as required by Dutch law and conform to the relevant institutional regulations of the Erasmus MC and Dutch legislation on animal experimentation. Permission was obtained under license number AVD101002015273.

For our experiments we started with a total of 115 mice, 5 of which were removed because of unreliable genotyping (1), early death (3), or illness as indicated by abnormal development and low weight (1). The *Emx1-Foxp2* group consisted of 18 wild type (9 female) and 9 homozygous knockout mice (5 female) from 3 litters; the *Rgs9-Foxp2* group consisted of 7 wild type (5 female) and 7 homozygous knockout mice (3 female) from 4 litters; the *L7-Foxp2* group consisted of 14 wild type (7 female) and 18 homozygous knockout mice (9 female) from 5 litters. Additionally, 15 mice (8 female) presented with no conditional knockout and a heterozygous spontaneous *Foxp2* mutation; this group will be referred to as *Foxp2-SD* (see Supplementary Fig. 4). Lastly, 37 mice had both a conditional knockout and a heterozygous spontaneous *Foxp2* mutation^56^; this group was excluded from all analyses. Knockout mice from the *Emx1-Cre Foxp2, Rgs9-Cre Foxp2* and *L7-Cre Foxp2* groups were only compared to wild type littermates that did not show spontaneous deletions. The precise genetic identity of *Foxp2-SD* animals was variable in that the spontaneous deletions probably occurred either in the germline or early during embryonic development, resulting in a heterozygous deletion for *Foxp2* in either a fully global or mosaic fashion, respectively. Mice with spontaneous deletion of *Foxp2* were identified with PCR using specific primers. Though the rate of spontaneous deletions in these mice is high, these occurrences are not unheard of^57,58^.

### Ultrasonic vocalization recordings

Ultrasonic vocalizations (USVs) were recorded from pups with the age of P5 (postnatal day 5), P7, P9 and P11. Litters were tested one at a time, with the pups tested in random order. Before recording, the litter was separated from the dam, and kept warm at a stable temperature using an infra-red lamp (Philips PAR38 IR 100 W E27). We separated the litter from the dam, as keeping the dam with the litter makes it harder to quickly and efficiently remove a pup from the litter, thus leading to an unnecessary increase in experimental variability^59^. Subsequently, the pup was placed in a small plastic container (6 × 6 × 5 cm) in a dark sound-proof box in which its USVs were recorded for 330 s with a CM16/CMPA microphone (Avisoft-Bioacoustics) that was positioned approximately 10 cm above the pup. The bottom of the container was lined with small rectangular cotton pads (Cotopads) to attenuate movement noise, and these pads were replaced with new ones after each recording. After recording, the pup was weighed and returned to the litter. All recordings were fed through an UltraSoundGate 116Hb interface (Avisoft-Bioacoustics) and digitized at 250 kHz with a bit-depth of 16 bits. The gain of the amplifier was kept at a level at which the loudest vocalizations rarely saturated.

### USV analysis

All recordings were analyzed with custom Python-based software. 5 min recordings were saved as WAV-files containing individual recordings were read using Scipy^60^. Data were subsequently high-pass filtered at 30 kHz using a 5th order Butterworth filter. After filtering, putative calls were identified by splitting the data into 256 sample fragments and assessing the cumulative power within those fragments using a threshold. This threshold was based on the sum of the median power of all fragments as well as the robust median deviation of all fragments. All threshold-passing fragments within 4096 samples were concatenated, as they presumably form a single, multi-fragmented call, such as a call featuring frequency jumps. The locations in time of these fragments were then stored and subjected to a Fast Fourier Transform (FFT) to determine whether the fragment contained a call or noise. A buffer of 2048 samples was added at the start and end of each fragment to reduce the risk of losing fainter parts of the vocalization. FFTs were then run on the data with a window size of 512 samples (75% overlap), which results in a temporal resolution of 0.512 ms, given a 250 kHz sampling rate. As the Nyquist frequency of the data was 125 kHz, the frequency resolution of the resulting FFT was 244.14 Hz.

To determine the pitch, duration and number of segments in a vocalization we analyzed the FFT data using techniques from image processing. First, an array containing a single vocalization was dilated (4 iterations) and subsequently eroded (4 iterations) to merge segments that were initially artificially segmented. The fragment was considered to contain an actual USV if at least one segment was longer than 4 ms, and if the number of segments did not exceed 10. After this, the components of the vocalization were identified and labeled, and the duration and pitch of each segment was measured. Each segment was also fitted by a 5th-order polynomial in order to facilitate later categorization and reproduction. Next, each vocalization was categorized as one of only three shapes; each vocalization was either categorized as "short" (i.e., under 5 ms), as "simple" (i.e., containing a single segment), or as "complex" (i.e., comprising of multiple segments, containing e.g., multiple harmonics or frequency jumps). We preferred to only use three categories so as to reduce the odds of false positives, as large numbers of variables require large numbers of statistical analyses (for other categorization schemes, see e.g., Refs.^17,19,61^).

In addition, we analyzed so-called "clicks". We define clicks as short (1 ms), broadband noises predominantly in the infrasound frequency range (up to approximately 50 kHz). These clicks have been identified sporadically in earlier research of *Foxp2* mutant mouse pups^20^ but have not been investigated in detail. To detect these clicks we performed high-pass and low-pass filtering of the recordings at 5 kHz and 30 kHz, respectively. We then identified all 256 sample fragments that had a cumulative power over a set threshold. Clicks that lasted longer than 1.5 ms or that had another above-threshold fragment within 5 ms of its centre were excluded for analyses, as these clicks tended to be artificial due to noise, such as movement of the pup. As the analysis to extract the clicks was low-pass filtered at 30 kHz, all USVs were effectively removed from the data; the click analysis was thus completely independent from the USV detection algorithm, in that clicks are detected with no prior knowledge of the temporal location of USVs.

Using the extracted data, we calculated a group of variables for each recording. First, we calculated the rates of USVs, clicks, and events (i.e., the sum of USVs and clicks), all converted to occurrences/minute. Second, we analyzed the median vocalization pitch and mean vocalization duration. Third, to investigate the impact of respiratory behaviour we analyzed the vocalization data using parameters based on Sirotin et al.^37^. In this study, the authors found that USVs that occur within 60 ms of each other are vocalizations uttered in the same respiration. We thus analyzed the number of USVs and clicks within 60 ms of each other, and we calculated the percentage of respirations that contained more than a single vocal emission. In addition, we defined a bout of vocalizations as a single vocalization-containing respiration, or a sequence of vocalization-containing respirations, with the maximum inter-respiration interval set at 300 ms. We calculated the average lengths of these bouts, and the percentage of bouts longer than a single respiration. We considered a vocalization-containing respiration to be a singular unit to increase clarity concerning the nature of a bout (e.g., whether a bout that is two events long is a single respiration with two calls, or two respirations with one call each). These parameters allow us to define whether a mouse predominantly vocalizes in sequences or in isolated events. As vocalizations and clicks often co-occurred, we omitted all clicks that co-occurred with USVs for the quantification of event and click rates as well as the calculation of respiration- and bout-sequencing. The rationale for this decision was that we were interested in the sequencing of clicks as independent units. After categorization, all events and metadata were saved in an SQLite database. Additionally, all the polynomial fit parameters for all segments of the calls were saved in a separate table. A visual representation of the analysis of the data can be found in Fig. 1.

### Statistics

All data in the figures are represented by a boxplot, the middle line of which indicates the median, the top and bottom edges indicate the 75th and 25th percentile, respectively, and the whiskers indicate 1.5 × IQR. Group means, medians, and IQRs, as well as the results of all statistical analyses are listed in Supplementary Table 2. All data-analyses were done using R^62^ and Python. For each group, 12 parameters were analyzed in total: USV rate (Hz), click rate (Hz), call rate (Hz), mean USV length (ms), median USV pitch (Hz), weight (g), percentage USVs over 75 kHz (%), percentage of respirations with more than one USV (%), percentage respiration with more than one click (%), percentage respirations with more than one call (%), percentage of bouts with more than one USV-containing respiration (%), percentage of bouts with more than one click-containing respiration (%), percentage of bouts with more than one call-containing respiration (%), For the analysis of the various parameters per group (e.g., *Rgs9-Foxp2, Foxp2-*SD) we used various mixed effects models. More precisely, for normally distributed, continuous dependent variables we used the lmer function from the lme4 package^63^. For Poisson distributed data we used the glmer function from the same package, unless the data displayed overdispersion, in which case the glmmTMB from the glmmTMB package was used^64^ with the nbinom2 family specification. If the dependent variable was proportion data, then it was transformed using the logit function. All mixed effects models were fitted with mouse_id and litter as random factors. To assess significance, we applied a log likelihood ratio test using the anova function in R. We compared, in order, the model with only random factors, a model with random factors and age, a model with random factors, age, and genotype/spontaneous deletion, and lastly a model with random factors, age, genotype/spontaneous deletion and the interaction of the fixed factors. As we assessed 12 parameters for each group, all p-values were corrected by Benjamini–Hochberg FDR correction, using SciPy^60^. All p-values in text and in figures are post-correction p-values. To assess the inter-event interval distributions, we used a glm model with time and vocalization-type as independent variables and proportion of total vocalization count per time-bin as the dependent variables. We used the bs function from the splines package to introduce knots at 10, 60, 100, 220, 300 and 400 ms, to allow for a better fit with the multimodal IEI distribution.

To determine whether the co-occurrence of clicks and USVs was more prevalent than expected if the clicks were distributed randomly, we calculated the overlap between clicks and USVs for all wild type animals. Then, for each recording, we randomized the location of the clicks and calculated the proportion of clicks overlapping with USVs. We repeated this process 100 times per recording, and calculated the average proportion of overlapping clicks/USVs for each of these recordings. These values were then compared to the actual overlap values of these recordings using a dependent t-test.

For the testing of event rates, click rates and USV rates, all available data of all mice were used. For the tests of sequencing characteristics involving USVs, such as the percentage of respirations containing more than one USV, and the percentage of bouts longer than one USV-containing respiration, we excluded the data of mice that emitted fewer than 5 USVs in total.

The possible effects of gender on the dependent variables were assessed for each group (i.e., *Emx1-Foxp2, Rgs9-Foxp2, L7-Foxp2, Foxp2-SD)*. We found no significant effects of gender, nor did we find a significant effect of the interaction of gender with age or the interaction of gender with the other independent between-subjects variables (i.e., either genotype or presence of a spontaneous deletion). All results were thus based on data from all genders pooled together.

## Supplementary Information

Supplementary Information.

Supplementary Table 1.

Supplementary Table 2.

## Data Availability

The data that support the findings of this study are available upon reasonable request.
