# Supplementary material for: Region-specific Foxp2 deletions in cortex, striatum or cerebellum cannot explain vocalization deficits observed in spontaneous global knockouts
Source: Sci Rep. 2020 Dec 10;10:21631. doi: 10.1038/s41598-020-78531-8 (PMC7730140; doi:10.1038/s41598-020-78531-8)
Supplement: Supplementary file 1 — Supplementary Information. [file 41598_2020_78531_MOESM1_ESM.docx]

**Supplementary Figures and Table legends**

Region-specific *Foxp2* deletions in cortex, striatum or cerebellum cannot explain vocalization deficits observed in spontaneous global knockouts

# Bastiaan H.A. Urbanus^1*^, Saša Peter^1^, Simon E. Fisher^2,3^ & Chris I. De Zeeuw^1,4*^

1 Department of Neuroscience, Erasmus MC, 3000 DR, Rotterdam, The Netherlands.

2 Language and Genetics Department, Max Planck Institute for Psycholinguistics, Nijmegen, The Netherlands.

3 Donders Institute for Brain, Cognition and Behaviour, Radboud University, Nijmegen, The Netherlands.

4 Netherlands Institute for Neuroscience, 1105 CA, Amsterdam, KNAW, The Netherlands.

* Correspondence: b.urbanus@erasmusmc.nl and c.dezeeuw@erasmusmc.nl


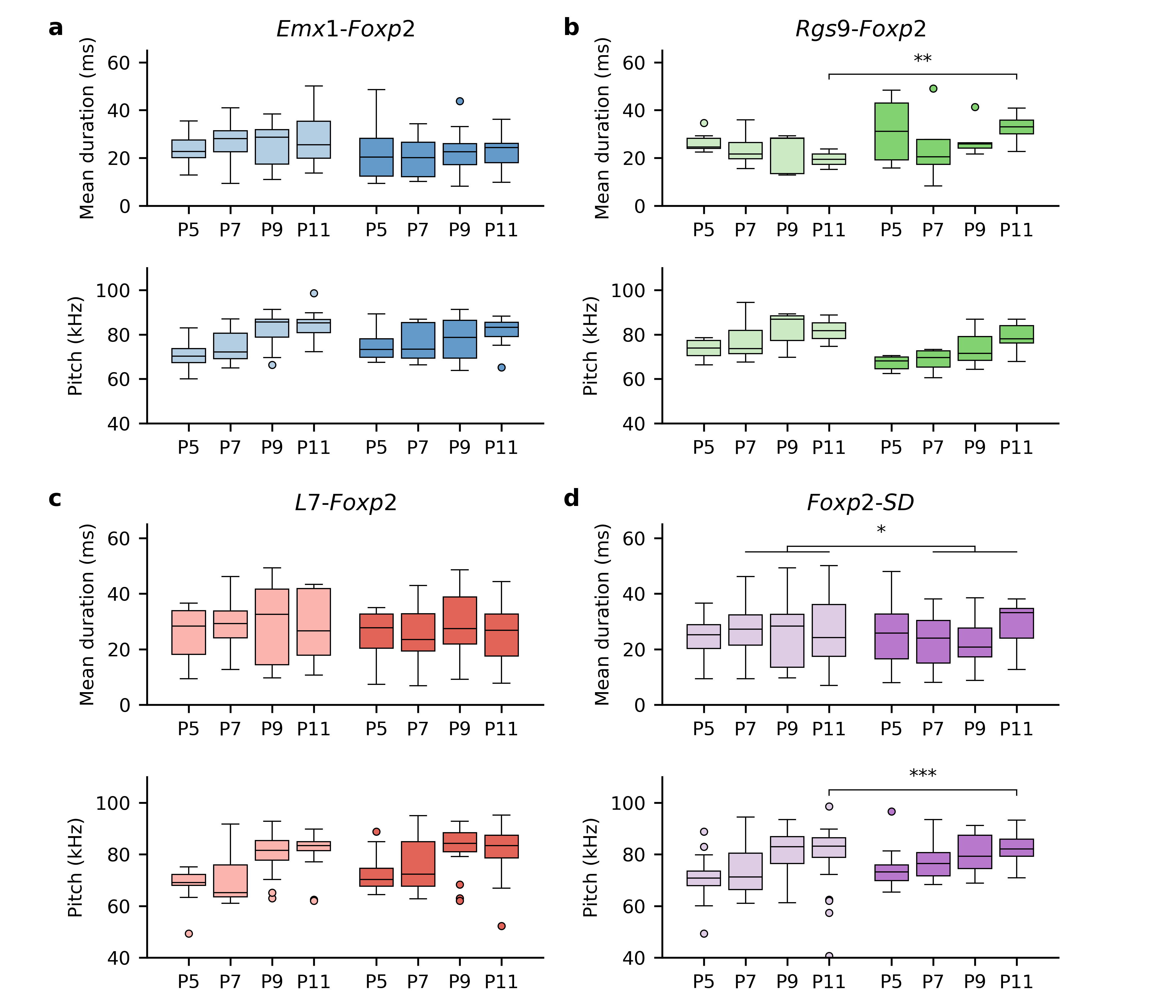
**Supplementary Figure 1:** USV duration and pitch are mostly unaffected in the three conditional knockout lines.

(**a**) Neither mean vocalization duration nor median vocalization pitch are affected in the *Emx1-Foxp2* mice. (**b**) Mean vocalization duration is increased in the *Rgs9-Foxp2* mice at P11 (top). Pitch is unaffected (bottom). (**c**) Neither vocalization duration nor vocalization pitch is affected in the *L7-Foxp2 mice*. (**d**) Vocalization durations are affected at P7, P9 and P11 in the *Foxp2-SD* animals (top). Moreover, the median pitch of USVs is affected at P11. Significance: * = *p* < 0.05; ** = *p* < 0.01; *** = *p* < 0.001.


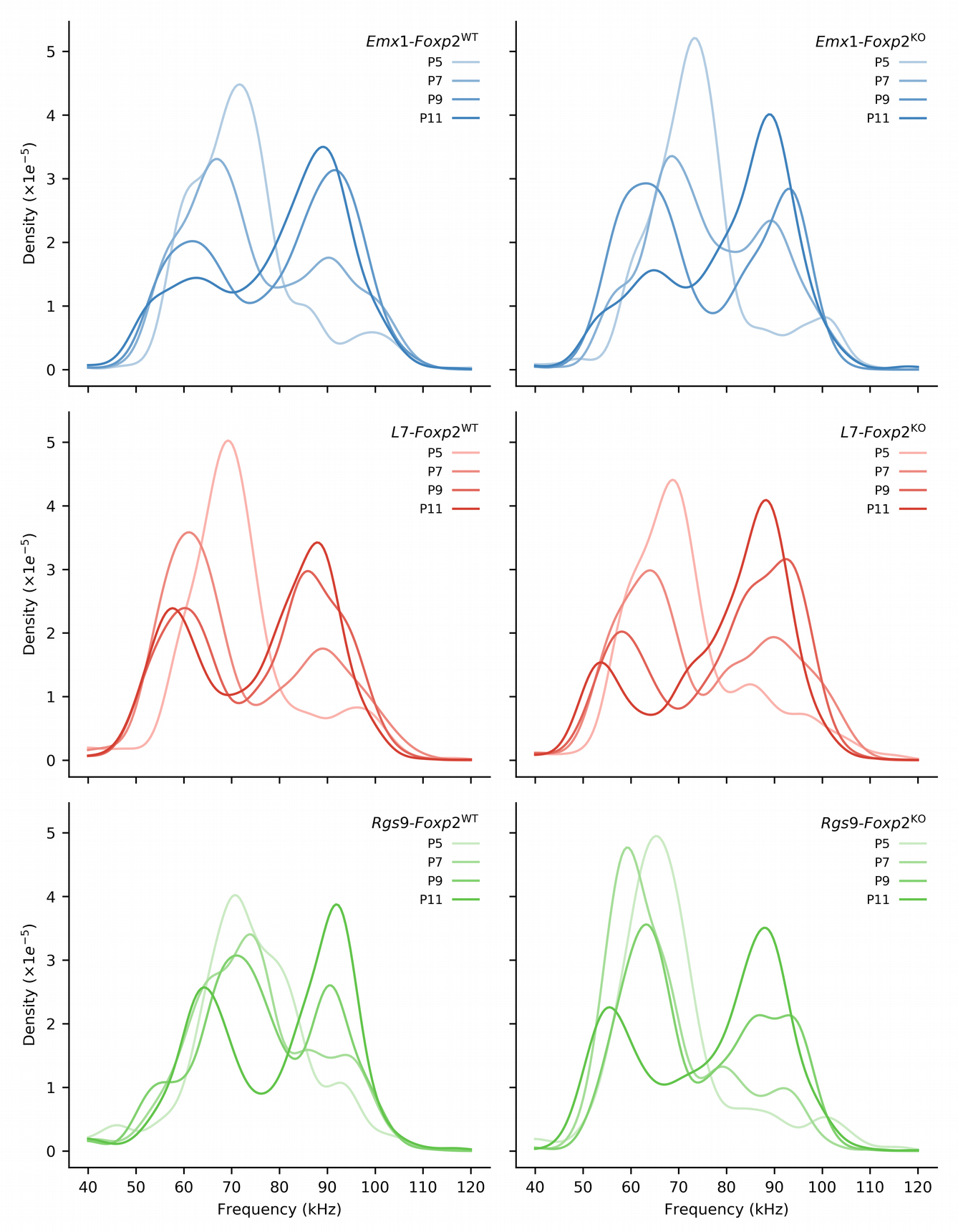
**Supplementary Figure 2:** Both WT and conditional knockout mice from all groups show a consistent shift from low to high pitched vocalizations over time.

Vocalization pitch is distributed bimodally in pups, with one peak between 60 and 70 kHz, and one peak around 90 to 100 kHz. The switch from a preference for lower vocalizations to a preference for higher pitched vocalizations is gradual, and happens in all groups. Y-axis scaling is constant for all subfigures.


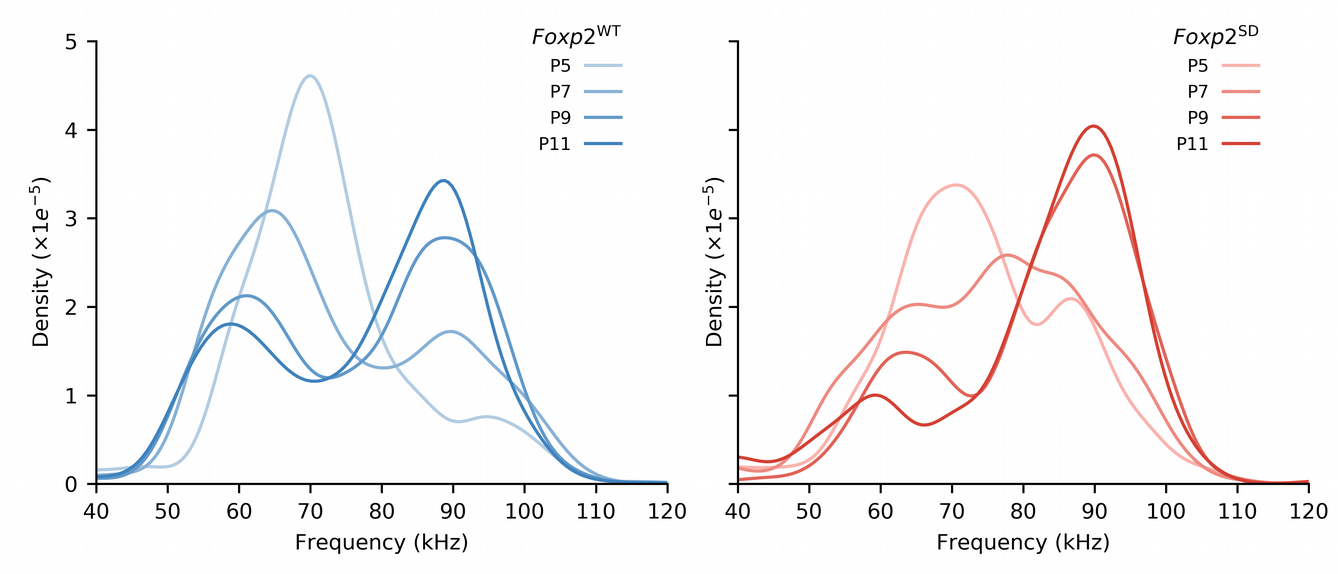
**Supplementary Figure 3:** USV pitch development is unaffected in *Foxp2-*SD mice.

Consistent with the pitch development in healthy mice, vocalization pitch moves from approximately 60 kHz at P5 to 90 kHz at P11 in the *Foxp2-*SD mice. The distribution is somewhat less defined, which is possibly due to the large reduction in the number of USVs emitted by these mice. However, the overall shift in pitch-preference is consistent over time. The Y-axis scale is the same for both figures.

**
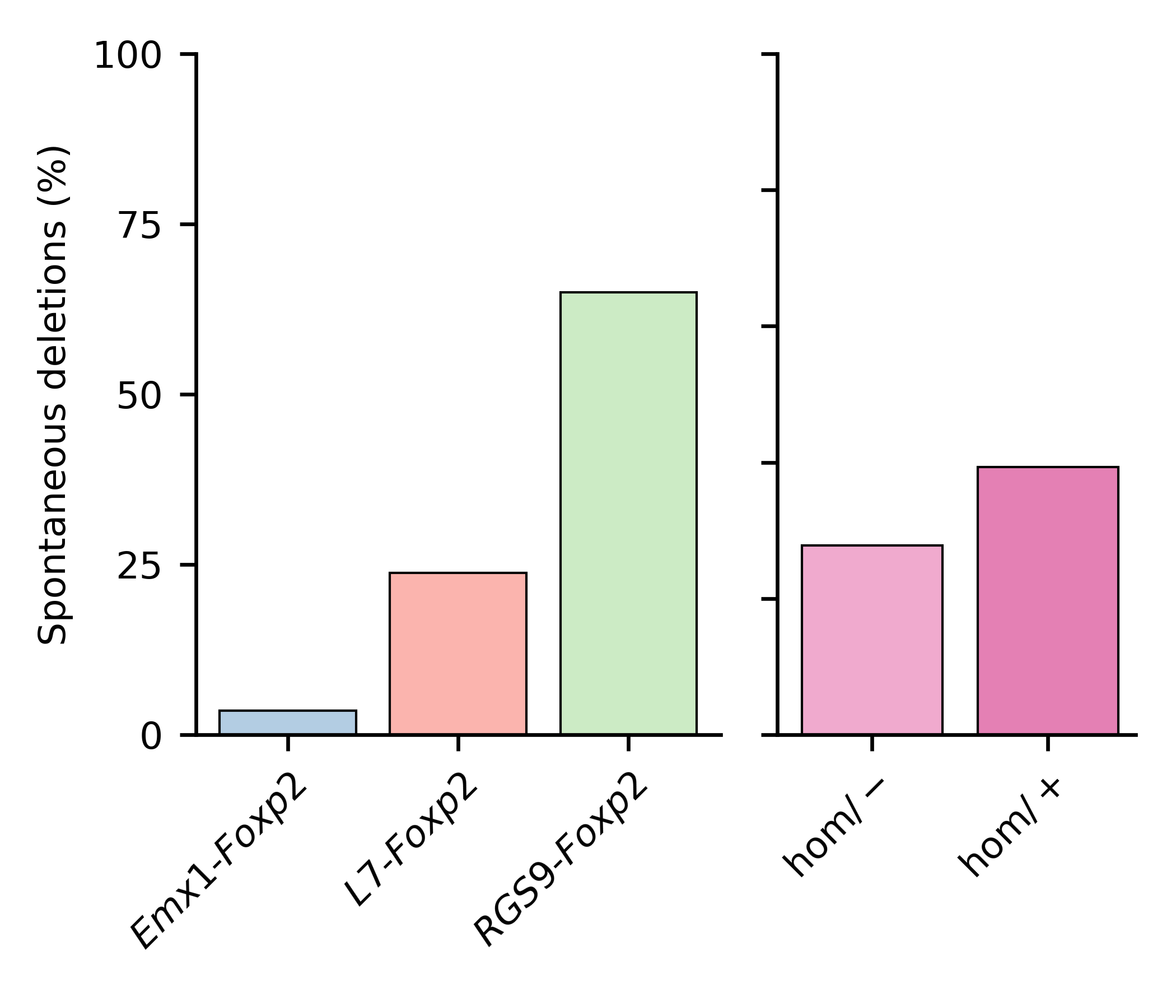
**

**Supplementary Figure 4:** The three conditional knockout lines present with varying numbers of spontaneous *Foxp2* deletions.

The percentage of animals with spontaneous deletions for each conditional gene knockout group, and the rate of spontaneous deletions for WT animals and mutant animals from the three groups combined. Y-axis scaling is constant for both the left and right plot.

**Supplementary Table 1:** Overview of primers used for the various conditional *Foxp2* KO lines.

**Supplementary Table 2:** Overview of statistical analyses used, by conditional *Foxp2* KO line, as well as an overview of the means of each group at each time point.

“emx1”, “l7”, and “rgs9” contain the results of all statistical analyses for the *Emx1/Foxp2*, *L7/Foxp2* and *Rgs9/Foxp2* conditional KO lines. The “rgs9-late” sheet contains the statistical analyses for the truncated *Rgs9/Foxp2* dataset, using only the data from P9 and P11. The “Medians” sheet contains all medians, means, sample sizes and standard deviations for all tested variables at all tested ages for each genotype. The “IEI” sheet contains the statistical analyses performed for the investigation of click and USV inter-event intervals.
